# Quantification of Appetitive and Mechanical Aversive Associative Learning Paradigms in *Drosophila* via a Y‐maze Assay

**DOI:** 10.1002/cpz1.70385

**Published:** 2026-05-15

**Authors:** Samuel J. Mabry, Thaanvi Malgireddy, Sean Sarkissian, David P. Saleeby, Zachary Freyberg

**Affiliations:** ^1^ Department of Psychiatry University of Pittsburgh Pittsburgh Pennsylvania USA; ^2^ Department of Cell Biology University of Pittsburgh Pittsburgh Pennsylvania USA; ^3^ These authors contributed equally to the manuscript

**Keywords:** associative learning, Drosophila, memory, odor conditioning, Y‐maze

## Abstract

Learning and memory are essential for the acquisition, storage, and recall of information, enabling organisms to adapt to changes in their environment. Associative learning begins when a stimulus, either positive or negative in valence, is presented alongside a neutral stimulus. This drives the development of an association between the stimuli, leading to the subsequent establishment of a learned behavioral response to the previously neutral stimulus. Associative learning is well conserved across species, from invertebrates to humans, and is highly amenable to experimental manipulation. *Drosophila melanogaster* is a valuable model for dissecting the mechanisms underlying associative learning and memory, given its genetic tractability, low cost, and the high‐throughput nature of fly behavioral assays. Here, we describe two distinct associative learning paradigms in the *Drosophila* model that employ olfaction to establish short‐term learned associations using a Y‐maze assay. We first describe a novel negative associative learning paradigm employing a mechanical aversive stimulus. We next describe a positive associative learning paradigm in which flies learn to approach an odor paired with an appetitive sucrose reward. Overall, these protocols offer an inexpensive and streamlined approach to investigating associative learning under a multitude of different contexts. © 2026 The Author(s). *Current Protocols* published by Wiley Periodicals LLC.

**Basic Protocol 1**: Negative associative learning paradigm utilizing mechanical stimulation

**Basic Protocol 2**: Positive associative learning paradigm utilizing sucrose feeding

## Introduction

Learning and memory are essential for the acquisition, storage, and recall of vital information that enables successful adaptation to environmental changes. Integral to these processes is associative learning, which occurs when a stimulus, either positive or negative in valence, co‐occurs with an otherwise neutral stimulus. Consequently, this pairing leads to an association in which the previously neutral stimulus becomes a cue for either a positive or a negative outcome. Importantly, associative learning is highly conserved across species, including in *Drosophila melanogaster* (Yamamoto & Seto, 2014). Due to this conservation, *Drosophila* have been used for decades as a robust model system to probe the neural mechanisms underlying associative learning (Beck et al., 2000; Felsenberg et al., 2017; Quinn et al., 1974; Waddell et al., 2000). *Drosophila* offer significant experimental advantages, including short generation times, genetic tractability, low maintenance costs, and amenability to conducting high‐throughput behavioral experiments. Just as importantly, findings in the fly can often be readily translated to mammalian models.

Extensive research has previously established that the *Drosophila* mushroom body (MB) is an integrative brain region critical for associative memory formation (Adel & Griffith, 2021; Cognigni et al., 2018). Distinct families of neurons (*e.g*., dopamine neurons) project to different regions of the MB and can individually associate either a positive or a negative valence stimulus with an odor, thereby forming new associations and updating previously learned ones (Otto et al., 2020). Fly olfactory conditioning paradigms have demonstrated that flies can form associations between odor cues and stimuli, enabling quantitative measurement of associative learning and memory acquisition (Beck et al., 2000). This is exemplified by the Y‐maze conditioning paradigm, which is based on classical conditioning and in which the fly learns to associate an odor with either an appetitive or an aversive stimulus (Mohandasan et al., 2022; Simonnet et al., 2014). While the Y‐maze assay has been used for decades, we aimed to develop a versatile, inexpensive, and readily accessible version of it.

Here, we describe high‐throughput, cost‐efficient methods for probing short‐term aversive and appetitive associative olfactory memories in *Drosophila*. We use a novel mechanical aversive stimulus to elicit avoidance behavior in Basic Protocol 1, and an appetitive sucrose reward to elicit approach behavior in Basic Protocol 2. Notably, these approaches are adaptable to other paradigms, such as aversive foot shock or additional appetitive food rewards. Moreover, the protocols can be easily combined with binary expression systems (*e.g*., UAS/GAL4) to permit robust testing across various genotypes/gene manipulations and experimental conditions.

*NOTE*: All protocols involving animals must be reviewed and approved by the appropriate Animal Care and Use Committee and must follow regulations for the care and use of laboratory animals. Appropriate informed consent is necessary for obtaining and use of human study material.

## Basic Protocol 1 NEGATIVE ASSOCIATIVE LEARNING PARADIGM UTILIZING MECHANICAL STIMULATION

We present a detailed protocol for establishing a negative associative learning paradigm in the *Drosophila* model. The workflow consists of three main sections: (A) preparation of the flies for the assay, (B) establishing conditioning via odor pairing, and (C) testing in the Y‐maze and associated data collection. Though we focus on mechanical stimulation as the aversive stimulus, these methods are meant to be generalizable and can therefore be adapted to other stimuli with negative valence. All assays are performed under light conditions during the flies' subjective day.

### Materials

**Figure 1:**
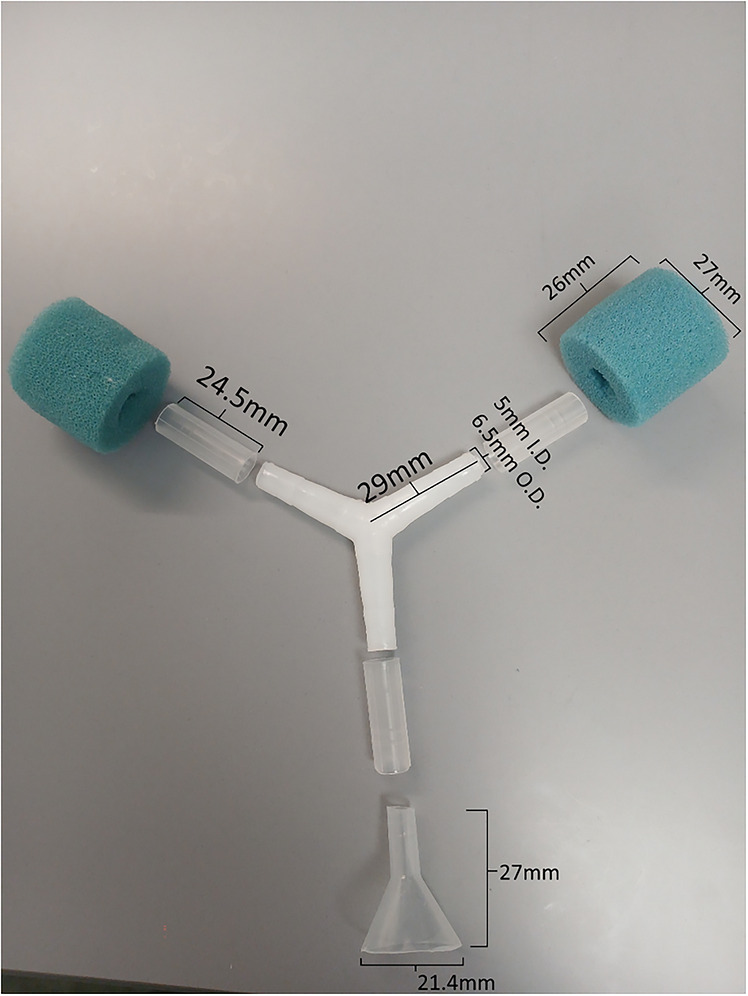
**Y‐Maze Apparatus Details**. Photograph and measurements of all parts of the Y‐maze apparatus, including tripartite connector, cut pipette tips, funnel, and sponge.

**Figure 2:**
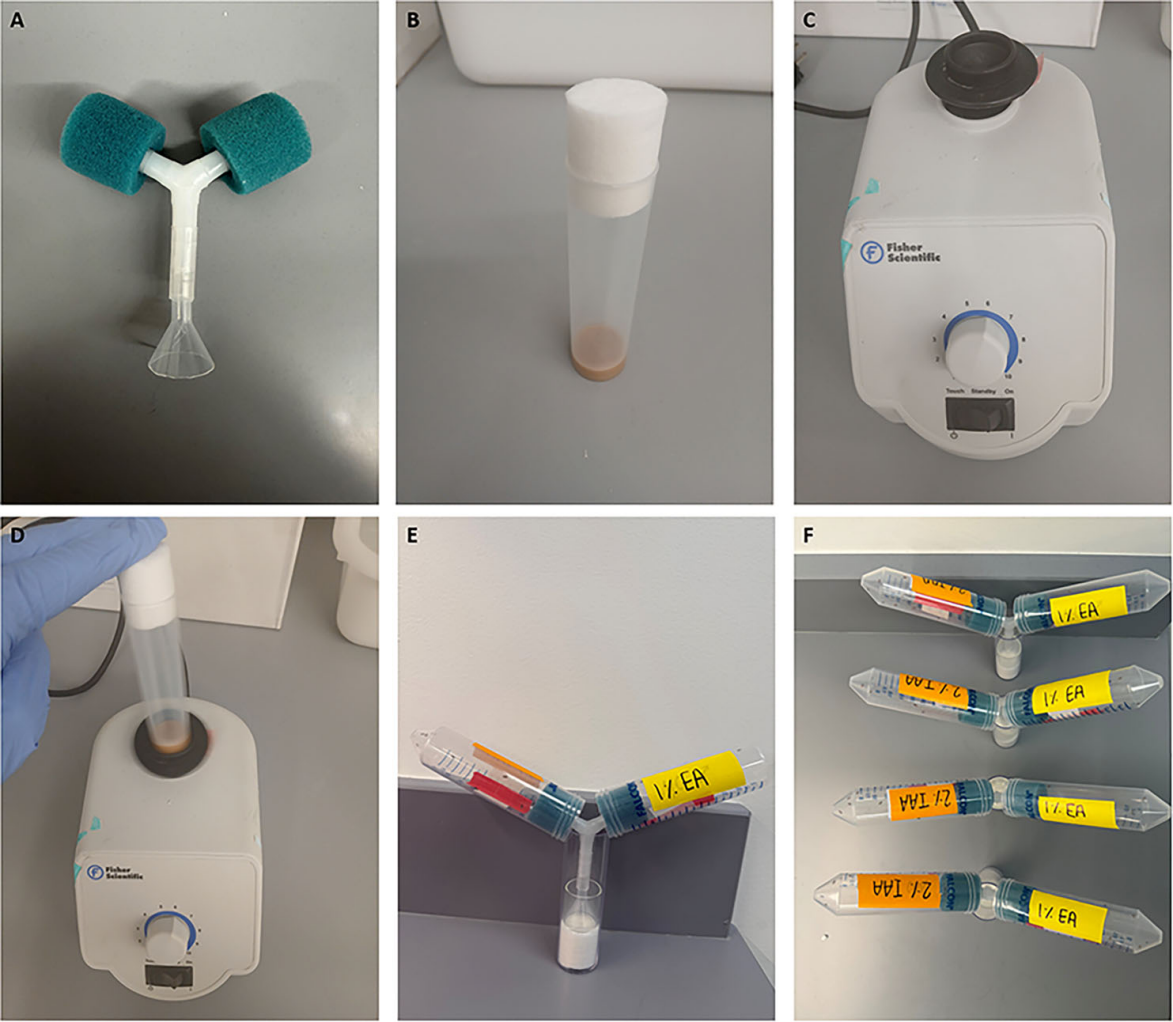
**Experimental Y‐maze setup. (A)** Photographs of the Y‐maze without attached conical tubes, funnel, and beaker. **(B)** Fly food medium in training vial. **(C)** Vortex for mechanical stimulation. **(D)** Vortex‐shaking a fly food medium vial. **(E)** Full Y‐maze apparatus with *Drosophila* choosing which odor‐associated arm to enter. **(F)** A 4‐fold Y‐maze apparatus running simultaneously.

Needed for both protocols
*Drosophila* of any genotype
  Polypropylene narrow *Drosophila* vials (Fisher Scientific #AS507)
*Drosophila* mouth pipette
  Flystuff *Drosophila* CO_2_ fly pad (Genesee Scientific #59‐114)
  Y‐maze apparatus (**Figure** **1**)
  20‐mL glass beaker (**Figure** **2**)
  50‐mL conical tubes
  Small plastic funnel (**Figure** **2**)
  Odors: Isoamyl alcohol (IAA, Millipore Sigma #8222551000) and Ethyl acetate (EA, ThermoFisher Scientific #031344.AP)
  Mineral oil (Sigma‐Aldrich #M5904)
  Filter paper (Fisher Scientific #09‐801‐AA)
  Agar (Sigma‐Aldrich #A9539)
  Fly media
  Timer
Needed only for Basic Protocol 1
  Analog vortex mixer (Fisher Scientific #9454FUALUS, **Figure** **2**)
Needed only for Basic Protocol 2
  Sucrose (Fisher Scientific #S5‐500)
  Microwave

#### Section 1: Preparation of flies

1 Collect adult flies (2‐6 days post‐eclosion).
2 Transfer 30–40 flies to each of the narrow polypropylene *Drosophila* vials using a mouth pipette. Do not use CO_2_ to anesthetize the flies, as it will impair brain function and, in turn, the assay.

#### Section 2: Conditioning/odor pairing

**Figure 3:**
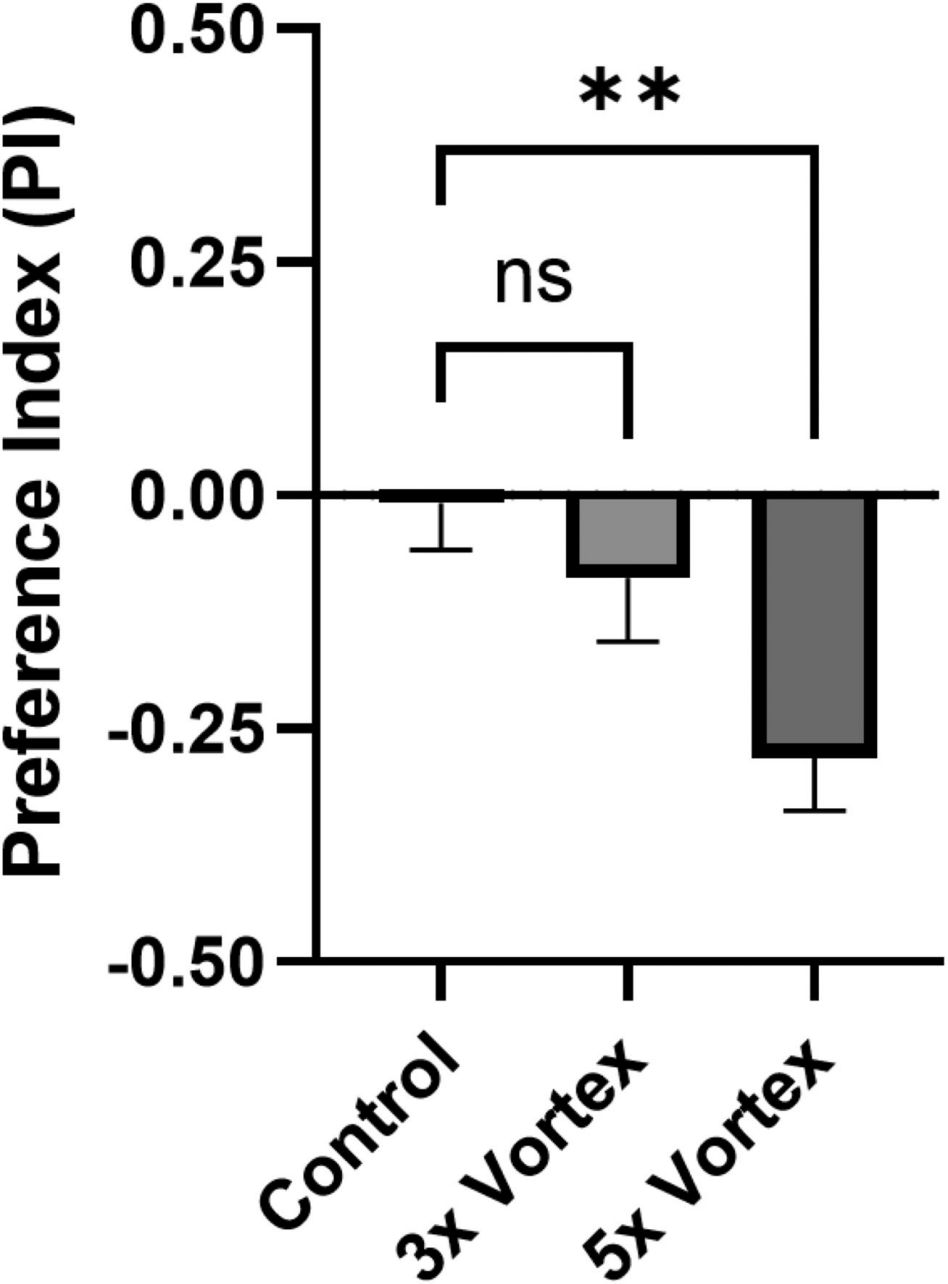
**Aversive olfactory associative learning paradigm utilizing mechanical stimulation**. Graph of preference index (PI) of wild‐type *Drosophila* after undergoing aversive olfactory conditioning with a mechanical vortex stimulus (*F*_2,48_ = 5.513, *p* = 0.0070, ** = *p* < 0.01).

3 Pipette 40 µL of the ethyl acetate (EA) or isoamyl alcohol (IAA) odor solutions (see Reagents and Solutions for details) onto filter paper and secure with tape inside the respective vials. Prepare all odor vials at least 1 h before continuing with the protocol.
4 Divide flies into balanced groups: half will be conditioned with IAA and half will be conditioned with EA.
5 Transfer flies into the control odor vial and let them rest for 5 min.
  EA for flies to be conditioned with IAA, and IAA for flies to be conditioned with EA.
6 Transfer flies from the control odor vial to a vial with no odorant for 1 min.
7 Transfer flies from the odorless vial to a vial containing the odor for which the flies will be conditioned.
8 Vortex flies for 15 s (medium speed, equivalent to a setting of 6 on the vortexer model used in this assay; see **Video** **1**), then allow flies to rest for 1 min. Repeat this 5 times. For the control condition, place flies into the odor vial for 5 min and do not vortex.
  This vortex stimulation can be repeated greater or fewer than 5 times, to elicit either a more or less robust memory, respectively. However, excessive vortexing (e.g., 10+ times) damages the flies and will prevent subsequent decision‐making (see section 3; **Table** **1**; **Figure** **3**).

**Video 1:** **Representative video for** **Basic Protocol** **1**. Flies were placed into a learning vial and gently vortexed for 15 s.

**Table 1:** Troubleshooting Guide for Establishment of Associative Learning Paradigms and Y‐maze Use

Problem	Potential Solutions
Flies failing to make any decisions in the Y‐maze by staying in the original compartment. If the assay is performed correctly, 80–90% of the flies should make a decision.	1) Ensure that the Y‐maze assay is performed with the apparatus positioned vertically. Flies have a natural inclination to climb up after being tapped to the bottom of the assay chamber. 2) In Basic Protocol 1, vortexing the flies at an overly high setting can cause them to fail to make a decision. Vortex on a moderate setting to avoid physically damaging the flies.
Flies failing to consume the food in Basic Protocol 2	1) If the flies fail to consume the food, a longer starvation period may be needed.
Flies failing to properly associate the stimulus with the desired odor	1) It is critical that the flies are not exposed to any other outside odors during the performance of the assay. Avoid using fragrances, including hand lotion, perfumes, or other odorous substances, on the day the assay is performed. 2) In Basic Protocol 1, the flies are mouth pipetted into the desired vials before the experiment begins. Perform mouth pipetting in a way that minimizes perturbing the flies. If flies sustain damage (*e.g*., broken wings) during this step, it can perturb the assay and elicit negative results.

#### Section 3: Y‐Maze testing and data collection

9 After conditioning, flip flies into a 20‐mL beaker and place into the Y‐maze apparatus (**Figure** **2**).
  The decision tubes are sealed at the ends to prevent flies from escaping after making a decision.
10 Allow flies to choose which arm of the Y‐maze to enter over a 10‐min span, timing this period with a timer.
  By the end of this 10‐min span, 80–90% of flies should have made a decision.
11 At the conclusion of the choice period, anesthetize flies with CO_2_ and record the number of flies in each odor arm, including the number of flies that failed to decide.
12 Calculate a preference index (PI): (Number of flies in paired odor arm − Number of flies in control odor arm)/(Number of flies in paired odor arm + Number of flies in control odor arm) (**Figure** **3**).

## Basic Protocol 2 POSITIVE ASSOCIATIVE LEARNING PARADIGM UTILIZING SUCROSE FEEDING

### Section 1: Preparation and starvation of flies

1 Collect adult flies (2–6 days post‐eclosion).
2 On the day prior to the assay, transfer 30–40 flies to each starvation vial using a mouth pipette or via CO_2_ anesthetization.
  Starvation vials are narrow polypropylene Drosophila vials with 1% agar at the bottom instead of standard fly food (See Reagents and Solutions for details).
3 Starve flies overnight (18–20 h).
4 On the day of the experiment, prepare 1 M sucrose‐supplemented fly medium and place this medium into narrow polypropylene *Drosophila* vials (**Figure** **2**). Prepare additional narrow vials containing fly medium without sucrose to serve as controls (See Reagents and Solutions for details).

#### Section 2: Conditioning/odor pairing

5 Pipette 40 µL of the EA or IAA odor solutions (See Reagents and Solutions for details) onto filter paper and secure with tape inside respective vials (either sucrose or vehicle fly media). Prepare all odor vials at least 1 h before continuing with the protocol.
6 Divide flies into balanced groups: half will be conditioned with IAA and half will be conditioned with EA.
7 Transfer starved flies into the control odor vial with vehicle food for 5 min.
  EA for flies to be conditioned with IAA, and IAA for flies to be conditioned with EA.
8 Transfer flies from the control odor vial into an odorless vial for 3 min.
  We use a slightly longer resting period between the CS+ and CS− odors in Basic Protocol 2, as this facilitates learning and therefore yields more consistent data within the assay.
9 Transfer flies from the odorless vial into a vial containing the odor for which the flies will be conditioned with 1 M sucrose media for 5 min. For the control condition, place flies into an odor vial with vehicle food rather than 1 M sucrose.

#### Section 3: Y‐maze testing and data collection

**Figure 4:**
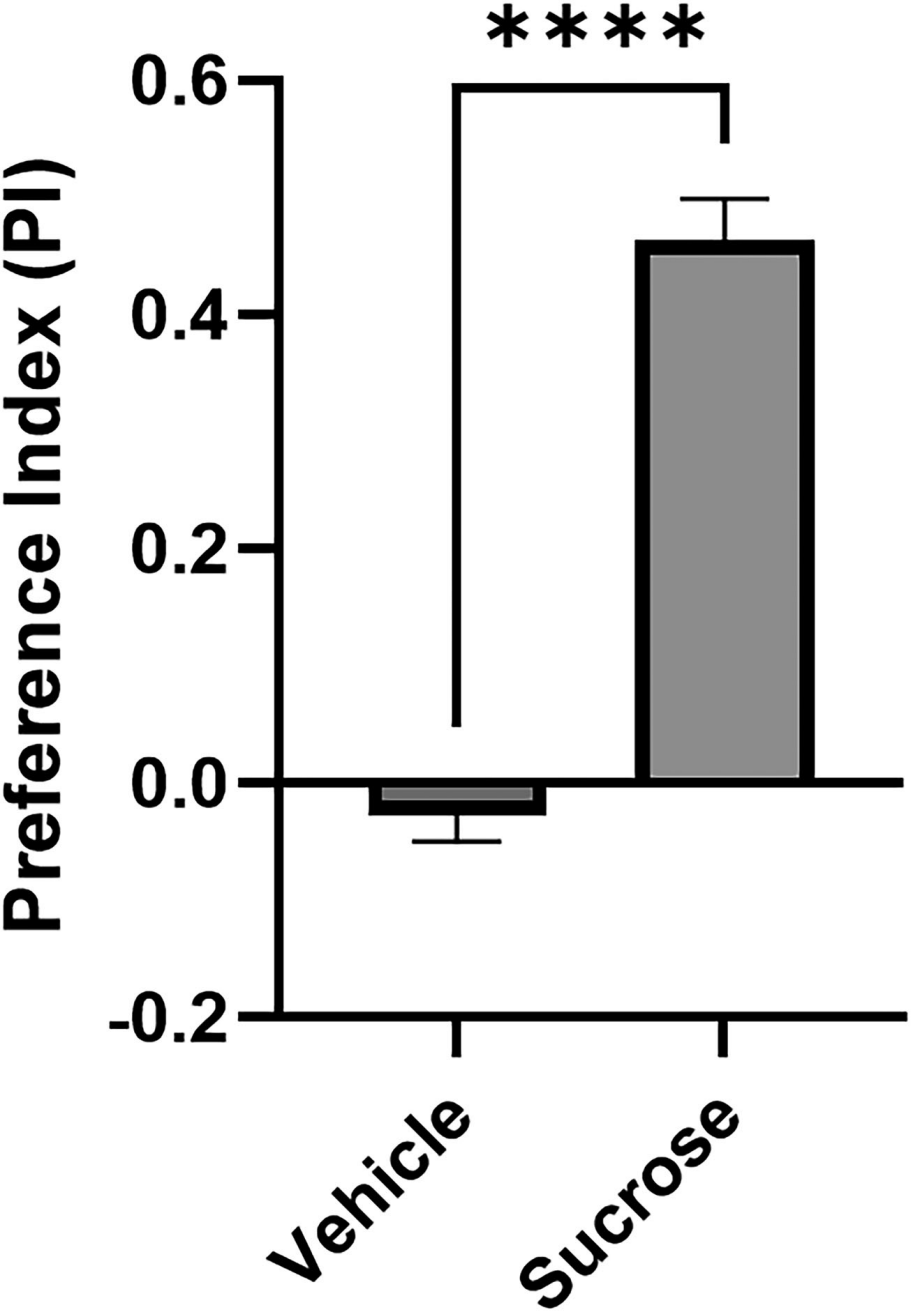
**Appetitive olfactory associative learning paradigm utilizing sucrose feeding**. Graph of preference index (PI) of wild‐type *Drosophila* after undergoing appetitive olfactory conditioning with 1 M sucrose (*t* = 8.925, *p* < 0.0001).

10 After conditioning, flip flies into the 20‐mL beaker and place into the Y‐maze apparatus (**Figure** **2**).
  The decision tubes are sealed at the ends to prevent flies from escaping after making a decision.
11 Time the flies for 10 min using a timer to allow flies to choose which arm of the Y‐maze to enter.
  By the end of this 10‐min span, 80–90% of flies should have made a decision.
12 Anesthetize flies with CO_2_ and record the number of flies in each odor arm, including the number of flies that failed to decide.
13 Calculate a preference index (PI): Same calculation as in Basic Protocol 1 (**Figure** **4**).

## Reagents and Solutions

Use double‐distilled, deionized water unless otherwise specified.

### Agar vials for starvation

Make a 1% agar solution in double‐distilled/deionized water. Boil until the agar is completely dissolved, then add 5 mL to a narrow polypropylene *Drosophila* vial and allow 1–2 h for the agar solution to fully solidify. Remove excess condensation from the inside of each vial before adding flies. If not used promptly, refrigerate immediately after solidifying and use within 1 week. Please do not use if the agar has dried and pulled away from the vial walls.

### EA Solution

Dilute EA in mineral oil at a 1:50 concentration. Make fresh before each experiment.

### IAA Solution

Dilute IAA in mineral oil at a 1:100 concentration. Make fresh before each experiment.

### Sucrose‐supplemented fly medium

Melt the fly food in the microwave for approximately 30 s. Allow the food to cool until it is warm to the touch, then add sucrose to the still‐liquid food to a final 1 M concentration. After mixing thoroughly, add 5 mL to each narrow polypropylene *Drosophila* vial and allow 1–2 h for the sucrose‐supplemented fly food to fully solidify (**Figure** **2**). Remove excess condensation from the inside of the vial before adding flies. Please use promptly within the same day.

## Commentary

### Background Information

*Drosophila* has commonly been used as a model of olfactory associative learning for decades (Beck et al., 2000; Felsenberg et al., 2017; Quinn et al., 1974; Waddell et al., 2000). While different associative learning paradigms are often tailored to examine specific questions, we purposely established this protocol to highlight the general principles of two fundamental versions of the paradigm: negative/aversive and positive/appetitive associative learning. Our aim is to make it more accessible for a broader audience of researchers to perform simple learning and memory assays in *Drosophila*. Consequently, we describe the Y‐maze odor‐associative learning assays, whose central advantages include low setup cost, ease of performance, and high‐throughput capability. Moreover, this assay is amenable to medium‐ to large‐scale forward genetic screens in *Drosophila*, enabling the discovery of novel gene modulators.

The techniques described here are also amenable to studies related to both short‐ and long‐term memory formation in *Drosophila*. Indeed, building on the short‐term assays outlined in Basic Protocols 1 and 2, repeated pairings with the same stimuli (vortex or sucrose) can drive longer‐lasting memories that can be tested with the same Y‐maze apparatus. Overall, these assays are indispensable tools for neurobiologists investigating associative learning and memory formation.

### Critical Parameters

#### Odor Pairing

Both Basic Protocols 1 and 2 are organized to ensure a defined order for conditioned odor and stimulus pairing. However, in our experience, the order of stimulus pairing does not significantly alter the outcome of the experiment. Ultimately, it is important to conduct both stimulus‐pairing orders as a control (e.g., pair the odor with the vortex first, then pair the second neutral odor). Furthermore, it is crucial to always split the trials so that half are associated with one odor (e.g., IAA) and the other half with the other (e.g., EA) to ensure no bias toward one odor over the other.

#### CO_2_ Anesthesia

It is important to never expose the flies to CO_2_ on the experimental day. CO_2_ and other anesthetics can drastically impair certain types of memory formation in *Drosophila*. If the flies have previously been exposed to CO_2_, it is best to wait 16–20 h after the last CO_2_ exposure to allow them to fully recover before performing a Y‐maze assay.

#### Starvation Length

For Basic Protocol 2, we have had the best results with an overnight (18–20 h) starvation before feeding flies 1 M sucrose‐supplemented fly food. However, for other sucrose concentrations or appetitive stimuli (e.g., alcohol, cocaine), different starvation durations may be necessary to achieve optimal results.

#### Funnel and Beaker Setup

It is important that the small plastic funnels fit into the 20‐mL beaker at the bottom of the Y‐maze apparatus so that no flies can escape through the edges. It may be necessary to slightly cut the plastic funnels to ensure an ideal fit.

#### Cleaning and reuse

All parts of the testing apparatus, including Falcon tubes and pipette tips, can be cleaned and reused between assays. Avoid cleaning with odorous substances (e.g., ethanol). Thoroughly rinse all parts of the apparatus separately and allow them to dry overnight before use.

### Troubleshooting

Refer to Table 1 for a list of potential problems and troubleshooting suggestions.

### Time Considerations

Basic Protocol 1 can be completed in one day once sufficient flies have been collected to perform the assay. The assay usually takes 3–4 h from start to finish when performing 8–12 trials.

Basic Protocol 2 takes two days to complete once sufficient flies have been collected for the assay. Day 1 involves preparing starvation vials and starving the flies, which requires 2 h to complete. Day 2 involves performing the Y‐maze assay and requires 3–4 h from start to finish when conducting 8–12 trials.

### Author Contributions

**Samuel J. Mabry**: Conceptualization; data curation; analysis; methodology; visualization; writing—original draft; writing—review and editing. **Thaanvi Malgireddy**: Conceptualization; data curation; analysis; methodology; visualization; writing—original draft, writing—review and editing. **Sean Sarkissian**: Data curation, writing—review and editing. **David P. Saleeby**: Data curation; writing—review and editing. **Zachary Freyberg**: Conceptualization; project administration; supervision; funding acquisition; writing—original draft; writing—review and editing.

### Conflict of Interest

The authors declare no competing financial interests or relationships.

## Data Availability

The data that support the protocols are available from the corresponding author upon reasonable request.
